# A Detailed Analysis of the Effect of Different Environmental Factors on Fish Phototactic Behavior: Directional Fish Guiding and Expelling Technique

**DOI:** 10.3390/ani12030240

**Published:** 2022-01-19

**Authors:** Jiawei Xu, Wenlu Sang, Huichao Dai, Chenyu Lin, Senfan Ke, Jingqiao Mao, Gang Wang, Xiaotao Shi

**Affiliations:** 1 College of Water Conservancy and Hydropower Engineering, Hohai University, 1 Xikang Road, Nanjing 210098, China; xujiawei928@163.com (J.X.); dai_huichao@hhu.edu.cn (H.D.); linchenyu@hhu.edu.cn (C.L.); maojq@hhu.edu.cn (J.M.); wanggangnanj@163.com (G.W.); 2 Key Laboratory of Integrated Regulation and Resource Development on Shallow Lakes, Ministry of Education, School of Environment, Hohai University, Nanjing 210098, China; Sangwenlu723@163.com; 3 Hubei International Science and Technology Cooperation Base of Fish Passage, China Three Gorges University, Yichang 443002, China; 15926989126@163.com

**Keywords:** phototaxis, rheotaxis, water temperature, light environment, community restoration

## Abstract

**Simple Summary:**

Environmental pollution and hydropower development have affected fish survival and caused the extinction of some fish populations and species. To understand the effects of various environmental factors on the behavioral profiles of fish, we established a novel experimental method to measure the sensitivity and phototactic behavior of *Schizothorax waltoni* to four light colors and two flow velocities at two temperatures under low light intensity. The results showed that *S. waltoni* preferred the four light colors in the order green, blue, red, and yellow. *Schizothorax waltoni* showed positive phototaxis in green and blue light but negative phototaxis in red and yellow light. The increased flow velocity intensified the positive and negative phototaxis of fish under different light environments, while an increase in the water temperature aroused the escape behavior. Thus, red or yellow light greater than the phototaxis threshold can be used to move fish away from dangerous areas such as high-turbulent flows or polluted waters, while green or blue light can guide them to safe environments such as fish passage entrance or ideal habitats. Finally, this study provides scientific evidence and application value for restoring fish habitats, fish passages, and fisheries.

**Abstract:**

Optimization of light-based fish passage facilities has attracted extensive attention, but studies under the influence of various environmental factors are scarce. We established a novel experimental method to measure the phototactic behavior of *Schizothorax waltoni*. The results showed that *S. waltoni* preferred the four light colors in the order green, blue, red, and yellow. The increased flow velocity intensified the positive and negative phototaxis of fish under different light environments, while an increase in the water temperature aroused the escape behavior. The escape behavior of fish in red and yellow light and the phototaxis behavior in green and blue light intensified as the light intensity exceeded the phototaxis threshold and continued to increase. Thus, red or yellow light greater than the phototaxis threshold can be used to move fish away from high-turbulent flows or polluted waters, while green or blue light can be used to guide them to fish passage entrance or ideal habitats. This study provides scientific evidence and application value for restoring fish habitats, fish passages, and fisheries.

## 1. Introduction

Water conservancy infrastructure plays an essential role in economic and social development, while barriers cut off the passage of migratory fish and destroy their natural habitat, leading to the massive degradation of aquatic habitats and extinction of aquatic species [1,2,3]. To manage critical ecological issues and alleviate the negative impact of the hydroelectric complex, river managers and engineers have built many artificial fish passages such as fishways, fish barges and transport systems, and fish lifts [4,5,6]. Considering the function of environmental factors involved in optic, flow, acoustic, and other fluvial contexts in regulating fish movement [7], the environment media-based solutions are promising for fish passage design, aquaculture, and other efforts of protection [8,9]. It should be highlighted that the fish populations upstream and downstream of a dam are vulnerable to the entrance of the turbine and the tail water of power stations (a high turbulence area) due to the impact of the discharge of power stations. Fish migration will consequently lack a suitable fish passage and reach suitable habitats. Therefore, we can guide the fish to a safe area in advance and keep them away from dangerous areas. Single-variable experiments to determine the relationship between environmental factors and fish behaviors have been conducted, but the effects of multiple factors remain less understood. Hence, more well-designed and accurate experiments are urgently needed to decode fish behaviors in scenarios with more than one factor.

Environmental factors play an essential role in the growth and physiology of fish [10,11]. The non-specific response of fish to the adverse stimulus of external environmental factors is called a stress response. The stress response is the abnormal state of fish when the tolerance of adverse environmental factors reaches or approaches the limit, such as increased activity, panic avoidance, and endless swimming. The avoidance response is one of the most common stress responses, which is manifested by changing the original behavior of swimming and leaving the area. Light, water flow, and temperature are considered the three main factors influencing fish behavioral responses [8,12]. For fish survival [13], light can enable the fish metabolic system to respond appropriately [14]. Fish can use the retina and extra-retinal photoreceptors to determine the light intensity and spectral changes [15,16]; furthermore, different species respond differently to light [17]. For example, barfin flounder (*Verasper moseri*, Jordan & Gilbert, 1898) show a higher growth rate under short wavelength light (blue light) [18], and haddock (*Melanogrammus aeglefinus*, Linnaeus, 1758) have a higher frequency of predation behavior and catch more prey in blue light than in the light of other colors [19]. Generally, fish have low predation efficiency and survival rates in environments with high light intensity [20]. Environments with low light intensity support better initial development, higher survival, and a lower rate of intraspecific predation, in addition to reducing stress and stimulating growth [21,22]. Moreover, fish phototaxis, a typical response mechanism of aquatic animals to light stimulation, refers to the movement toward or away from the light source. Positive phototaxis refers to the movement toward the light source, while negative phototaxis refers to the movement away from the light source. When fish swim randomly, they lack phototaxis [23,24]. Therefore, suitable light can attract or repel fish and influence fish behavior [25].

Fish luring manipulation based on fish vision is applicable for various fish passages. For example, the lighting combined with the water charging system at the fish passage entrance can help fish locate the entrance quickly [26,27,28]. Additionally, proper lighting conditions improve habitat suitability and partially promote fish survival [29,30]. Collectively, studies on the behavioral response of fish to various light stimulation underlie the efforts in the recovery of the fish migration path and habitat restoration to some extent [8,31].

On the other hand, some studies have documented water flow as the predominant factor that governs fish swimming behavior [32]. Specifically, they have concluded that flow velocity might alter a fish’s heading strategy, swimming pattern, and ascending efficiency (the capacity to move upstream against the water current) [33]. Notably, water flow and light usually interact in the aquatic environment simultaneously, and thus, the effects of each factor cannot be analyzed in isolation. Fish migration, for instance, is significantly reduced in low-light environments [34]. Furthermore, the water in the background is generally flowing when river managers use light to guide fish toward safe areas (fish passage entrances or ideal habitats) or away from dangerous areas (high turbulent flows or polluted waters). Given the simultaneous presence and interaction of water flow and ambient light, disentangling the mechanisms of their superimposed effect on fish is necessary.

Besides light and water flow, water temperature is another crucial environmental factor that affects the survival and growth of aquatic organisms [35]. Water temperature below a certain limit favors aquaculture production, while the metabolic stress caused by temperature beyond the maximum limit adversely affects the growth, food consumption, and health of farmed fish [36]. For instance, juvenile sea bass grows fast at 22–25 °C, and their lethal temperature limits are at 2–3 °C and 30–32 °C [37]. The construction of hydraulic barriers substantially changes the water temperature in original rivers, with temperature variations of up to 3 °C daily and 12 °C yearly in the plateau area [38]. The discharge of cold water from dams affects aquatic biota at numerous levels, reducing their metabolic functioning, survival, growth, and the opportunity to spawn and recruit [39]. In addition, an increase in water temperatures increases stress in fish, forcing them to migrate upstream to more suitable waters, thus confining their habitat size [40]. Hence, understanding the behavioral responses of fish at different temperatures is also important for building fish passages, aquaculture, and habitat restoration.

This study concentrates on *Schizothorax waltoni* (family Cyprinidae, subfamily Schizothorax, genus Schizothorax, Regan, 1905), which is an economically and ecologically important cold-water species inhabiting canyon rapids and primarily distributed in the main tributaries of the middle reaches of the Yarlung Zangbo River in Tibet, China. Due to the long life span, adaptation to cold water, slow growth rate, and low fertility of the species, it is highly vulnerable to species invasion and human activities, such as overfishing and the construction of hydropower stations [41]. As a priority species for conservation, only a few attempts have been made to examine the impact of different environmental factors on the behavior of *S. waltoni*. To fully understand the behavior of *S. waltoni* under different environmental conditions, this study evaluates the behavioral response of *S. waltoni* under different light, water flow, and temperature conditions through a two-stage experiment. The study provides a scientific basis for optimizing various fish facilities, restoring fish habitats, and creating a suitable environment for the growth and development of fish.

## 2. Materials and Methods

### 2.1. Experimental Materials

Wild adult *S. waltoni* (mean ± SD; body length = 28.00 ± 3.60 cm; body mass = 245.00 ± 20.00 g) were sampled from the Yarlung Zangbo River, located downstream of the Zangmu hydropower station in Tibet, and temporarily reared in the fish breeding house of the power station camp. A total of 330 fish were involved in the experiment; 130 were used in circular tanks for the light sensitivity experiments whilst the remaining 200 were used in the rectangular flume experiments to test the influence of temperature and water velocity on the phototactic behavior of *S. waltoni*. Fish husbandry was conducted in circular tanks (2.50 m in diameter, 0.50 m in height, and 0.30 m in depth). The water used for cultivation and experimentation was kept at 15.00 ± 0.50 °C, and half of the water was replaced daily through a water pumping system connected to the river. We selected healthy individuals for the trials to ensure that the experiments were accurate. The criteria for selecting healthy fish were as follows: (1) There was no obvious trauma on the surface of the fish’s body. (2) The fish swam normally without floating on the surface of the water or turning the belly. (3) The color of the fish’s body did not change. (4) The fish swam in groups, were flexible and responsive, and dived into the water immediately after being frightened. Each individual was tested once when they could feed and swim normally after 48 h of fast.

### 2.2. Apparatus

**Figure 1:**
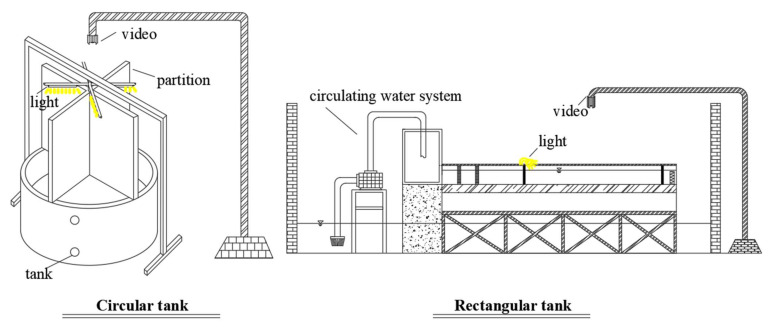
Experimental setup of the circular and rectangular tanks used to test the sensitivity of *S. waltoni* to different light colors (circular tank) and the influence of different water velocities and temperatures on the phototaxis of *S. waltoni* (rectangular flume). The circular tank was divided into four areas; different lights were placed in each area. The rectangular tank was separated by a transparent net into 2.20 m experiment areas. The light source was arranged upstream of the tank.

A circular tank was used to determine the sensitivity of *S. waltoni* to the light of different colors. The experimental apparatus comprised a light source, four baffles, and a monitoring system (Figure 1). The baffles divided the tank into four areas where the fish could swim freely, and a separate light source was installed above each area. We used an infrared camera (B12HV2-1, HIKVISON, Hangzhou, China) and a video recorder (DS-7800, HIKVISON, Hangzhou, China) to monitor fish behavior and used Tracker 6 for video analysis.

A rectangular flume (Experimental area: 2.20 m length × 0.55 m width) was used to examine the effects of different water velocities and temperature on the phototaxis of *S. waltoni*. The setup consisted of water circulation equipment, a rectangular tank, and monitoring equipment (Figure 1). We arranged two transparent nets on the upstream and downstream of the tank to isolate the experimental area, and derive a uniform and stable water flow through the water circulation system and the rectifier grid; the pump valve was used to maintain the water depth at 0.20 m.

### 2.3. Experimental Method

#### 2.3.1. Selection of the Color of Light

**Figure 2:**
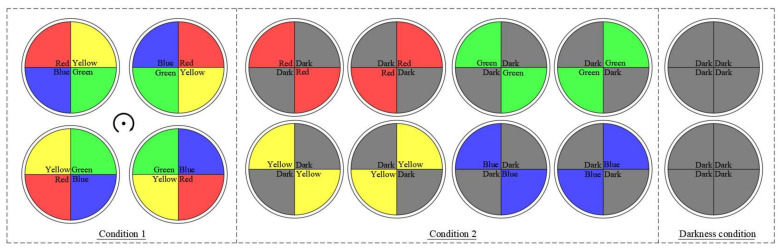
Selection of the color of light under experimental conditions.

This experiment aimed to observe the phototaxis of *S. waltoni* in red, yellow, green, and blue light environments and its preference and avoidance of the four light colors. The water temperature was checked before the experiment, and the light intensity was fixed in each light color at 10 lux. Before the experiment, a healthy individual of *S. waltoni* was placed in the middle of the circular tank, and the lights were switched on to start the experiment when the test fish could swim freely in the dark environment. Each experiment lasted one hour and was repeated ten times. The experiment was performed in two steps (Figure 2):

(1) The lights were arranged in the four areas of the circular sink in the order of red, yellow, green, and blue, and the phototactic behavior of the fish in each region was observed. After each trial, the original light color sequence was rotated clockwise to proceed to the next experiment.
(2) Four light colors were paired with darkness (which mimicked night-time conditions) and arranged in four regions; the light of the same color was arranged diagonally. The color was rotated clockwise at the end of each experiment, keeping the order unchanged to ensure that there is no interference with the location of the tank on fish selection during the experiment. An experimental treatment in the absence of light (dark treatment) was set up to compare and observe the preference and avoidance of the fish.

#### 2.3.2. Phototaxis Experiment

This experiment aimed to observe the effects of different flow velocities and water temperatures on the phototaxis of *S. waltoni* in four light environments. Before the experiment, a healthy individual of *S. waltoni* was placed in the middle of the experimental area (area 3). The light was switched on to start the experiment when the test fish was able to swim freely in the dark environment. Each experimental treatment was repeated 10 times.

**Figure 3:**
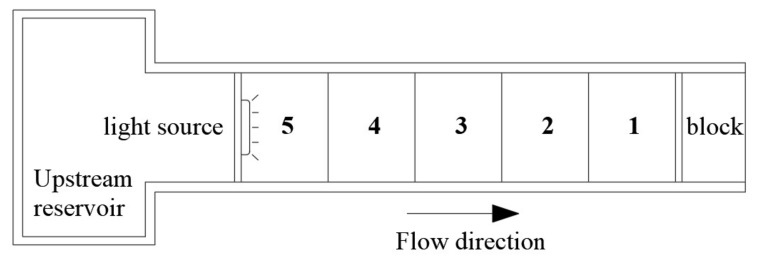
Top view of the rectangular experimental flume.

**Figure 4:**
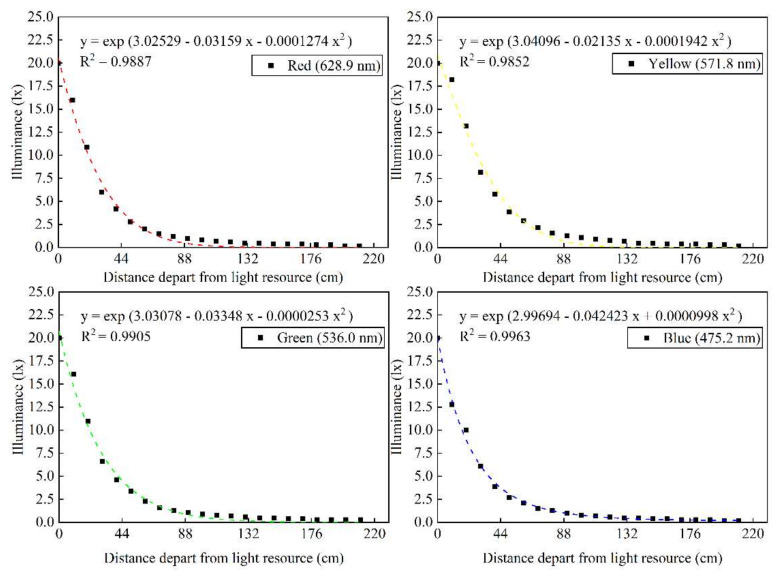
Optic attenuation of the light source used in the experiment.

The experimental area of the rectangular water tank was equally divided into five areas, which were marked one to five from back to front (Figure 3). At the top of the upstream (area 5), the light intensity was maintained at 20 lux, and the light intensity attenuated uniformly from areas 5 to 1. Light attenuation and flow direction were from regions five to one (Figure 4).

The experiments included two flow velocities (0 m·s^−1^ and 0.3 m·s^−1^), four colors (red, yellow, green, and blue), and two water temperatures (15 °C and 18 °C) as shown in Table 1.

### 2.4. Data Analysis

(1) We used the time proportion *F* to indicate the preference of *S. waltoni* in different environments:

$$F\%=\frac{f}{N}\times 100\%$$

In the equation, f is the distribution time of the fish in an area with a specific color of light; N is the total distribution time of the test fish.

(2) We used phototactic index (*R*) to demonstrate the positive and negative phototaxis of *S. waltoni* under different light environments:

$$R=\frac{L_{1}}{D_{2}}\times 100\%$$

In the equation, $L_{1}$ is the swimming time of the fish in the light area (areas 4 and 5), while $D_{2}$ is the swimming time of the fish in the dark area (areas 1 and 2); *R* = 1 indicates no phototaxis, *R* > 1 indicates positive phototaxis, and *R* < 1 indicates negative phototaxis.

(3) We used the selection index *E* to indicate the degree of preference of *S. waltoni* to the environmental factors. The equation eliminates the interference of different experimental flume structures on fish.

$$E=\frac{r_{ij}-P_{i}}{P_{i}}$$

$$P_{i}=\left(\overset{n}{\underset{j=1}{\sum }}r_{ij}\right)/N$$

In the equation, the selection index *E* = 0 indicates no preference, *E* < 0 indicates avoidance, and *E* > 0 indicates preference. A high absolute value indicates a stronger preference for environmental factors.

$r_{ij}$ is the time proportion of *S. waltoni* in the *i*th area with *j* environmental factors; $P_{i}\left(\mathrm{circular}\;\mathrm{tank}:\;i=1-4,\;\mathrm{Rectangular}\;\mathrm{tank}:i=1-5\right)$ is the proportion of *S. waltoni* that appeared in the four areas. To obtain the distribution probability $P_{i}$, we divided the time of fish distribution in a certain area by the total experimental time. *N* is the sum of the areas. The calculations are shown in Table 2.

### 2.5. Statistical Analysis

Analysis of variance was performed to determine the differences among the variables at different levels. Fisher’s LSD was performed for pairwise comparisons, and significant differences were considered at *p* < 0.05. Fish distribution trajectory were obtained by Tracker 6. Linear fitting was performed for the regression analysis to determine the correlation between variables. All data were processed in SPSS 24.0 (IBM, Armonk, NY, USA), and the results are presented as mean ± SE.

## 3. Results and Analysis

### 3.1. Sensitivity of S. waltoni to the Light of Different Colors

#### 3.1.1. Preference of the Light of Different Colors by *S. waltoni*

**Figure 5:**
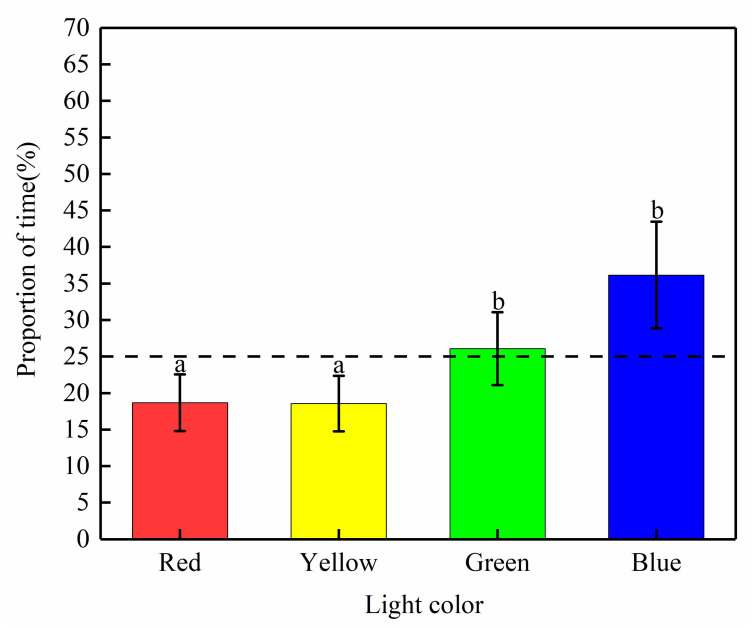
Time proportion of fish in the four regions with light of different colors. Different letters (a, b) associated with the bars indicate significant differences (*p* < 0.05).

*Schizothorax waltoni* showed a random swimming pattern in the four dark areas of the control treatment, with no significant difference in the distribution across the four areas, while test fish showed a significant difference in the distribution across the four light environments of red, yellow, blue, and green (Figure 5). Individuals of *S. waltoni* stayed in green and blue light longer than the average time (25%), and the time spent in the green and blue light areas was significantly higher than that in the red and yellow light areas (*p* < 0.05). The results indicated that *S. waltoni* prefers green and blue light.

#### 3.1.2. Determining the Phototaxis Behavior of *S. waltoni* in the Simulated Night-Time Environment

**Figure 6:**
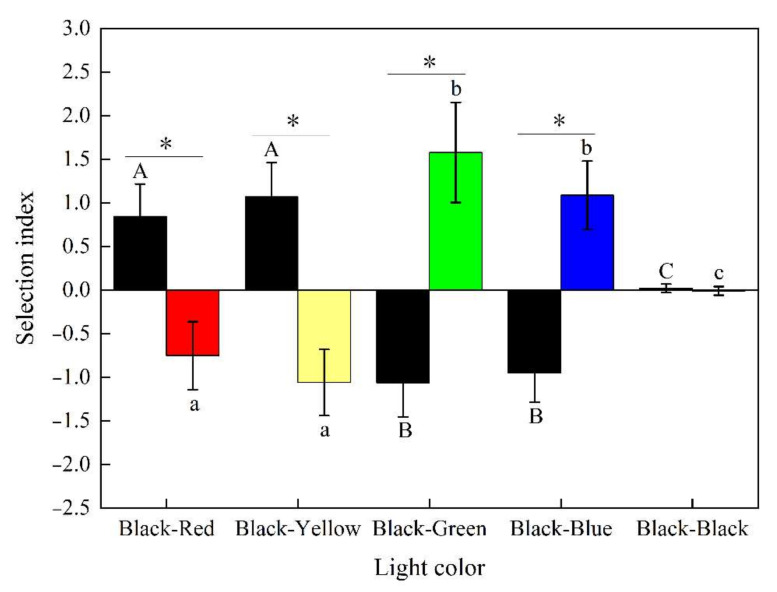
The selection index of the fish for five color combinations of light. Different capital letters (A–C) associated with the bars indicate significant differences among the dark groups (*p* < 0.05), different lowercase letters (a–c) associated with the bars indicate significant differences among light color groups (*p* < 0.05), and an asterisk (*) on the bars indicates significant differences within the group (*p* < 0.05).

The selection index of *S. waltoni* in five combinations of darkness and light environments is shown in Figure 6. The selection index of red and yellow light in *S. waltoni* was negative and significantly lower than that in the dark area (*p* < 0.05), i.e., the fish showed avoidance behavior in the red and yellow light areas. *S. waltoni*’s selection index in green and blue light was positive, and the absolute value was significantly higher than in the corresponding dark areas. (*p* < 0.05), indicating that the fish favored the green and blue light areas.

There was no significant difference in the choice of the area in total darkness (*p* > 0.05). The color preference of *S. waltoni*, determined from the selection index *E* of the different combinations, followed the order green > blue > red > yellow; the fish showed positive phototaxis to green and blue light, while they showed negative phototaxis to red and yellow light.

### 3.2. Behavioral Analysis of Water Flow Affecting the Light-Sensing Environment of Fish

**Figure 7:**
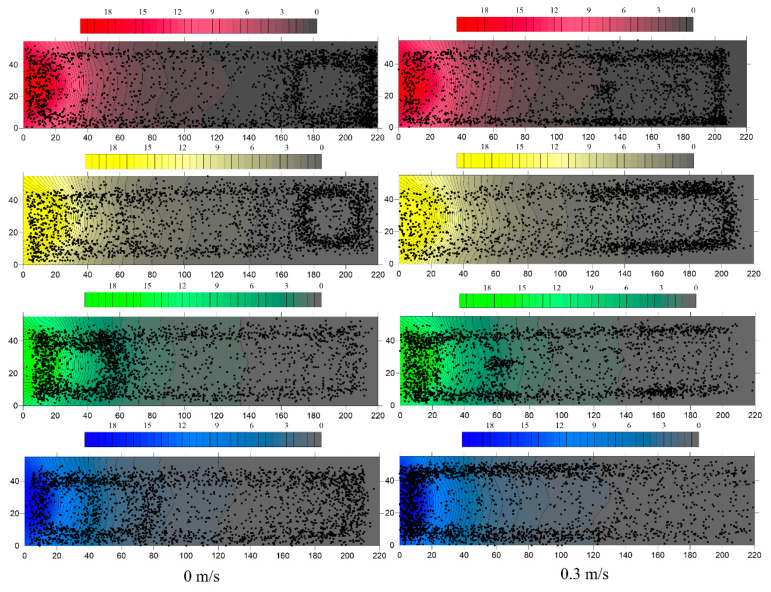
Distribution trajectories of the fish in red, yellow, green, and blue light at flow rates of 0 m/s and 0.3 m/s. The *y*-axis and *x*-axis are the length of the water tank in millimeters. The bar graph at the top represents the color change corresponding to the range of light intensity. The black points in the graph are the trajectories of the fish during the test period, and one black point represents one second.

The distribution trajectories of *S. waltoni* in the different colors of light under two flow conditions are shown in Figure 7. The individuals of *S. waltoni* continued to swim in different areas of the flume, but the trajectory proportions in the five areas under different light environments were different. In still water, the swimming trajectories of *S. waltoni* in red and yellow light were mainly distributed in the area far from the light source, and the swimming trajectories of green and blue light were mainly distributed in the area of the light source. The fish showed escape behavior in red and yellow light and approached green and blue light, which indicated that *S. waltoni* has negative phototaxis to red and yellow light and positive phototaxis to green and blue light. The fish swam between areas with different light intensities in different light environments and eventually showed “adaptive” rotational swimming. The light intensity corresponding to the circular trajectory (rotational motion) area in the figure is the light intensity range preferred by the fish in different light colors. Individuals of *S. waltoni* were found to swim toward the green and blue light sources (0–60 cm) and (0–70 cm), respectively, and rotate (170–220 cm) in the red and yellow light. Therefore, green and blue light at 20 lux attracted *S. waltoni*, while red and yellow light repelled them.

The distribution trajectories of *S. waltoni* at 0.3 m/s flow rate were similar to those in the still water treatment. *Schizothorax waltoni* moved away from the red and yellow light sources and toward the green and blue light sources. The positive and negative phototaxis light of *S. waltoni* under dynamic water conditions did not change, but its swimming behavior changed. *Schizothorax waltoni* did not rotate at 0.3 m/s, and its swimming trajectory was distributed at both ends and sides of the flume. The preferred area of *S. waltoni* under green and blue light changed (0–20 cm); the swimming trajectory was concentrated at the light source and concentrated in the dark area of the flume under red and yellow light. The results indicated that the behavior of *S. waltoni* in positive phototaxis light (green and blue light) and negative phototaxis light (red and yellow light) at 0.3 m/s flow rate was more conspicuous than in the static water conditions; in addition, the attraction to green and blue lights and the repulsion to red and yellow lights were strong.

The phototaxis index of *S. waltoni* in the light of different colors under two flow conditions is shown in Table 3. *Schizothorax waltoni* had the highest phototaxis index in still water under blue light, followed by under green light. The phototaxis index in red and yellow light was less than one, indicating that *S. waltoni* showed positive phototaxis to green and blue light and negative phototaxis to red and yellow light. In red and yellow light, the phototaxis index of *S. waltoni* at 0.3 m/s was significantly lower than that in static water (*p* < 0.05), while the phototaxis index of blue light was significantly higher (*p* < 0.05). Water flow can promote phototaxis of *S. waltoni*; in flowing water, positive phototaxis light can more effectively attract fish, and negative phototaxis light can more effectively drive fish away from the source of light.

### 3.3. Coupling Analysis of Rheotaxis and Phototaxis of S. waltoni at Different Water Temperatures

**Figure 8:**
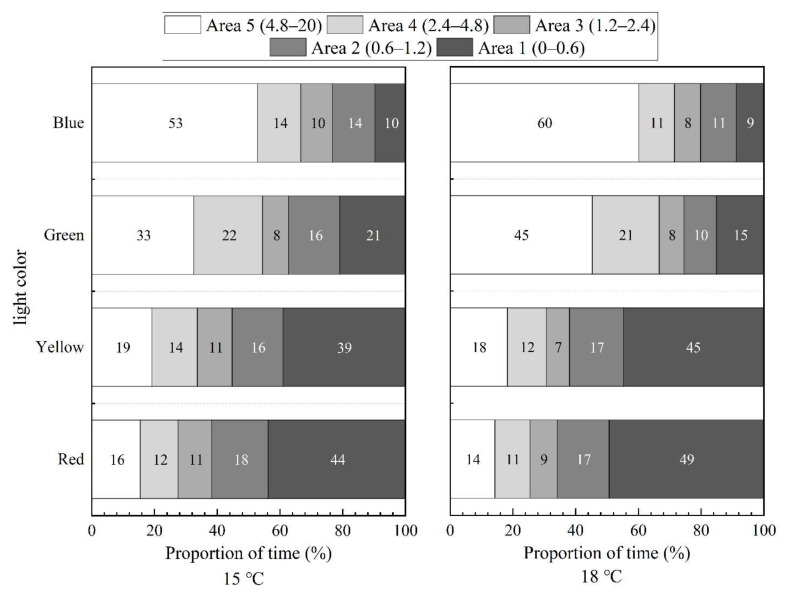
The time proportion of the fish in red, yellow, green, and blue light at a flow rate of 0 m/s. The numbers in the legend indicate the range of light intensity in each area. The numbers inside represent the average time proportions of the fish in different areas.

In still water, the distribution of *S. waltoni* fish under four lights (15 °C and 18 °C) is shown in Figure 8. Under red and yellow light, the time proportion of *S. waltoni* in area 5 was significantly higher than that in areas 1–4, and the time ratio in area 1 increased as temperature increased. *Schizothorax waltoni* moved away from red and yellow light sources. The fish preferred area 5 under green and blue light. The time proportion in area 5 increased with water temperature and decreased in other areas. The fish exposed to an abnormal water temperature (18 °C) were stressed and showed escape response; the direction of escape was consistent with the preferred area under different colors of light.

**Figure 9:**
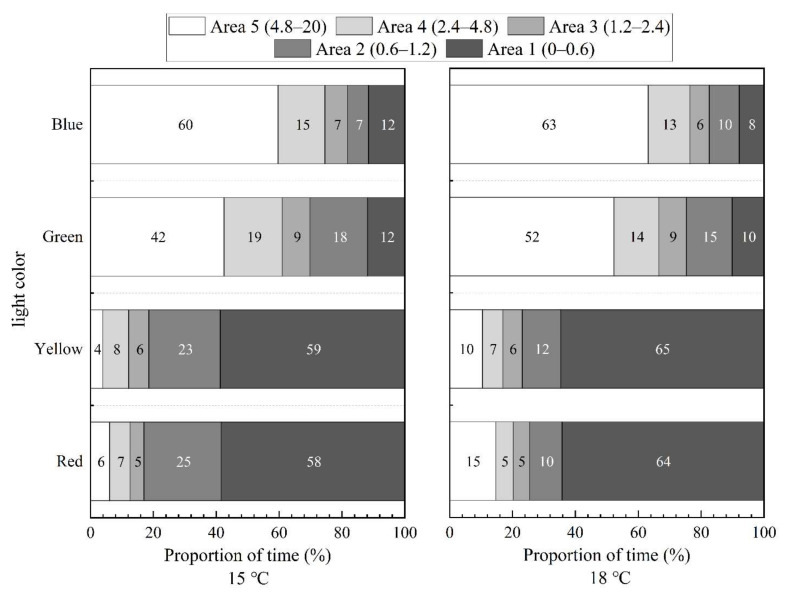
The time proportion of the fish in red, yellow, green, and blue light at a flow rate of 0.3 m/s. The numbers in the legend indicate the range of light intensity in each area.

The distribution of *S. waltoni* in red, yellow, green, and blue light (at 15 °C and 18 °C) and at a flow rate of 0.3 m/s is shown in Figure 9. Like their distribution in static water, *S. waltoni* was mainly distributed in the dark areas under red and yellow light and the bright areas under green and blue light. Additionally, the time proportion in area 5 under green and blue light increased with the increase in temperature and decreased in areas 1–4. Unlike in still water, in the red and yellow light, not only did the time proportion of fish in area 1 increase with the increase in temperature, but the time proportion in area 5 also increased. Thus, the fish were affected by the water flow, and they not only moved to the area of preferred light but also swam upstream to escape. In summary, the individuals of *S. waltoni* were uncomfortable in the water at 18 °C, and the abnormal temperature affected the regular behavior of the fish. The rheotaxis of the fish promoted their positive phototaxis behavior to some extent.

### 3.4. Phototaxis Threshold of Fish in Different Light Environments

**Figure 10:**
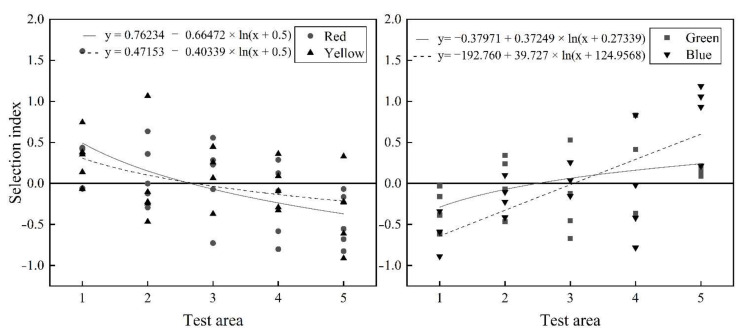
The selection index of the fish under red, blue, yellow, and green light in still water.

The test area (light intensity) and selection index were selected for functional regression analysis (Figure 10). The selection index trend line of *S. waltoni* in red light and yellow light showed a gradual decline with the increase in light intensity, i.e., the fish avoided the light source. The selection index of *S. waltoni* under green light and blue light showed a gradually increasing trend with an increase in light intensity, i.e., their preference increased with an increase in the proximity to the light source. The trend line in the figure shows that the selection index is zero from 2.5 to 3.0 for the red and yellow light, 2.0 to 2.5 for the green light, and 3.0 to 3.5 for the blue light. Therefore, the threshold for the red and yellow lights are 0.7–1.0 lux and 0.9–1.3 lux, while the thresholds for green and blue light are 0.6–0.8 lux and 1.0–1.7 lux, respectively. When the light intensity exceeded the light threshold, as the light intensity increased, the avoidance response of *S. waltoni* to red and yellow light and the preference for the blue and green light increased. The absolute value of the selection index in area 5 was the highest, and hence, in the study, green and blue light of 20 lux was used to induce, and red and yellow light of 20 lux was used to drive the *S. waltoni*.

## 4. Discussion

### 4.1. Selection of the Color of Light by S. waltoni

In this study, fish moved toward blue and green light and avoided red and yellow light. Additionally, their residence time in the blue and green light area was significantly greater than that in the red and yellow light area. Green and blue are positive phototaxis lights for *S. waltoni*, while red and yellow are negative phototaxis lights.

Fish can recognize the color of light and have different preferences for different colors [42]. The results of this study indicated that the order of preference for the four colors was: green > blue > red > yellow. By analyzing their behavioral responses in different light environments, it was found that *S. waltoni* swam continuously and tried to swim to the area under different light environments. *S. waltoni* showed a random swimming pattern in the dark environment and had no preference for any area, but they clearly preferred red, yellow, green, and blue environments. *S. waltoni* showed sudden U-turn avoidance or accelerated stress response to escape from an area when swimming through the red and yellow light environment. However, they stayed in the blue and green light environment and swam freely in those areas. After adapting to the blue and green light environment, the fish tried to swim to other areas, which could be related to the “signal” received by their photoreceptors. The sensitivity of the photoreceptors in fish adjusts to the wavelength of light in the ambient environment [43].

Differences in the habits and developmental stages of fish are the main reasons for the differences in phototaxis. The spectral composition of incident light changes differentially in underwater environments. Light attenuates rapidly with increasing depth, where the high-energy short wavelength (blue) end of the visible spectrum becomes predominant in deeper waters, while the low-energy red light penetrates only shallow waters [44]. Barfin flounder (*Verasper moseri*) showed a higher growth rate when farmed in short wavelength light, such as blue light [18]. Atlantic cod (*Gadus morhua*, Linnaeus, 1758) prefer to live in waters with shorter wavelengths (blue and green) of light than in waters with longer wavelengths (red) [44]. However, young taimen (*Hucho taimen*, Pallas, 1773) prefer a yellow environment, and zebrafish (*Barchydanio rerio var*, Goodrich and Nichols, 1931) prefer red light [45,46]. Barfin flounder and Atlantic cod live in deep waters, while the Hucho taimen and zebrafish live in surface waters. Hence, the barfin flounder and the Atlantic cod prefer blue–green light, while Hucho taimen and zebrafish prefer red and yellow light. *S. waltoni* is a demersal fish that lives in deeper water; this research shows that it prefers blue and green light, which is consistent with the preference of the above four species for light color, proving that the natural habits of fish species can affect phototaxis.

### 4.2. Preference of Light Intensity in S. waltoni

Luminosity is an essential environmental parameter for fish survival [13]. The manipulation of light intensity can regulate biological rhythms and physiological functions of fish, such as survival [47], growth [48], activity [49], feeding [50], and aggressive behavior [51,52]. Generally, the swimming style of fish changes across the day with the change in the ambient light intensity [53]. The survival and predation rates of nocturnal fish are low under high light intensity. Their feeding and aggression behaviors occur under low light intensity and are triggered by releasing chemicals such as melatonin [54]. Low light intensity may increase the release of melatonin, thereby improving fish activity. Therefore, low light is favored by fish. For example, halibut (*Hippoglossus stenolepis*, Temminck & Schlegel, 1846) has a phototaxis intensity range of 1–10 lux. When the light intensity exceeds this range, the fish exhibit an escape stress response and migrate to a low-light area. According to Boeuf et al. [24], low light critically affects the survival of fish. *Schizothorax waltoni* is found in Tibet, where the light intensity changes dramatically between day and night. Analyzing its behavior in a low light environment can help to understand its feeding and growth efficiency and attract the fish to habitats of interest or nutritional benefit via a perception-oriented fish sorting system [55]. Creating a weak light environment artificially can improve the quality of fish habitats.

In the current study, *Schizothorax waltoni* swam randomly in total darkness without a stress response. They approached the light source under green and blue light, and continued to swim around the light source (20 lux), but escaped from the area of the red and yellow light source and swam in the dark area (0.2–0.6 lux). *Schizothorax waltoni* exhibited rotational motion in four light environments. Individuals were found close to the green and blue sources of light, but they tried to swim to other areas and turn around upon reaching dark areas. However, they occasionally tried to approach the light source in the red and yellow light environment, and turned around and returned to the dark area when they reached the bright area and continuously rotated in the dark area. These behavioral characteristics confirmed that *S. waltoni* could recognize the color of light and were sensitive to light intensity. In this study, the fish were exposed to a strong light source and continued to move forward after acclimation. Under intense light stimulation near the light source, the fish lost their balance and returned to the dark area. The constant back and forth movement between low light and intense light to adapt is a pathological rotation motion observed around the light source [24,56]. Based on the above observations, we can artificially create a robust light environment to restrict fish from entering dangerous areas and construct a weak light environment to help them to grow and develop more efficiently.

### 4.3. Influence of Water Temperature on Phototaxis and Fluidity of Fish

Water temperature is an important environmental factor that affects the survival and behavior of fish. Different fish have different tolerances to temperature, and excessively high or low temperatures can cause the extinction of fish [57]. In this study, by determining the effects of different temperatures on the phototaxis and rheotaxis behaviors of *S. waltoni*, it was found that at a constant abnormal water temperature (18 °C), fish continued to show stress response and tried to escape from the test area. In still water, the escape direction was consistent with the area preferred by *S. waltoni* when exposed to the light of different colors. Fish escaped to the preferred area and tried to escape along the direction of the water flow affected by flow tendency under hydrodynamic conditions. An increase in water temperature accelerates the metabolic rate and respiratory rate and increases fish oxygen consumption rate, producing a stress response [58]. Our study suggested that, at 18 °C, blue and green lights strongly attract fish, while red and yellow lights repel them. Thus, at different temperatures throughout the day, green and blue lights can be used to attract *S. waltoni*, while red and yellow lights can be used to drive them away.

### 4.4. Interaction between Phototaxis and Fluidity

It is vital to choose the entrance position and shape of the fish collection system in the fish passage facility. The most common method of collecting fish is by using water flow to attract the fish. The entrance of the fish collection system is often arranged near the spillway or the power generation tailwater of a hydropower station to take advantage of the lure effect of the discharge of the hydropower station [59]. Further, by changing the shape of the fishway entrance to improve the flow pattern to attract fish [60], this technology can also be combined with light trapping technology. For example, the second hydropower station of the Bonneville Dam used light to attract fish into a bypass fish collection system [61]. Although many methods have been implemented to improve the effectiveness of fish facilities, the overall efficiency of the integrated fish luring measures is not ideal, which might be due to the lack of understanding of the interaction between phototaxis and fluidity.

Our research indicated that the phototaxis and fluidity of fish do not simply promote each other. As velocity increased, the phototaxis index of *S. waltoni* increased in green light and blue light but decreased in red light and yellow light. The results suggested that the phototaxis of *S. waltoni* to lights of different colors does not increase with the increase in water flow velocity, which might be related to the positive and negative tendencies of light and the amount of energy. This study found that flow velocity did not promote positive phototaxis of *S. waltoni* in red and yellow lights, but the increased flow velocity increased the negative phototaxis in red and yellow light. Hence, red or yellow light greater than the phototaxis threshold (light intensity) can drive fish away from dangerous areas, such as high turbulent flows or polluted waters, while green or blue light can guide them to safe environments, such as fish passage entrance or ideal habitats. In summary, this study provided scientific evidence and application value for restoring fish habitats, the passage of fish, and maintaining fisheries.

## 5. Conclusions

*S. waltoni* preferred the four light colors in the order green, blue, red, and yellow. *Schizothorax. waltoni* showed positive phototaxis in green and blue light but negative phototaxis in red and yellow light. The increased flow velocity intensified the positive and negative phototaxis of fish under different light environments, while an increase in the water temperature aroused the escape behavior. Specifically, the flow velocity of 0.3 m/s and temperature of 18 °C increased the attraction of *S. waltoni* to green and blue light and repulsion to red and yellow light.

The phototaxis thresholds of the test fish in the given four light colors were 0.7–1.0 lux (red), 0.9–1.3 lux (yellow), 0.6–0.8 lux (green), and 1.0–1.7 lux (blue). The escape behavior of fish in red and yellow light and the phototaxis behavior in green and blue light intensified as the light intensity exceeded the phototaxis threshold and continued to increase. Thus, red or yellow light greater than the phototaxis threshold can be used to move fish away from dangerous areas, such as high turbulent flows or polluted waters, while green or blue light can be used to guide them to safe environments such as fish passage entrance or ideal habitats.

## Figures and Tables

**Table 1:** Testing conditions of phototropism.

Temperature/Velocity	0 m/s	0.3 m/s
15 °C	Red	Red
	Yellow	Yellow
	Green	Green
	Blue	Blue
18 °C	Red	Red
	Yellow	Yellow
	Green	Green
	Blue	Blue

**Table 2:** The probability *P*_i_ of the distribution of *S. waltoni* in different areas in a circular and rectangular flume.

Circular flume	Area 1 $\left(P_{1}\right)$	Area 2 $\left(P_{2}\right)$	Area 3 $\left(P_{3}\right)$	Area 4 $\left(P_{4}\right)$	
	24.32%	17.33%	25.62%	32.73%	
Rectangular flume	Area 1 $\left(P_{1}\right)$	Area 2 $\left(P_{2}\right)$	Area 3 $\left(P_{3}\right)$	Area 4 $\left(P_{4}\right)$	Area 5 $\left(P_{5}\right)$
	37.26%	18.55%	6.61%	11.64%	25.94%

Areas 1–4 in the circular tank are arranged in the order of quadrants 1–4. In the rectangular water tank, areas 1 to 5 are arranged in the order of increasing light intensity, in which area 1 has the smallest light intensity and area 5 has the largest. *P_i_* (circular tank: *i* = 1–4, Rectangular tank: *i* = 1–5) is the proportion of *S. waltoni* that appeared in the four areas.

**Table 3:** Phototactic index of fish under different flow velocities and light environments.

	Red	Yellow	Green	Blue
0 m/s	0.45 ^a^ ± 0.35	0.61 ^a^ ± 0.41	1.46 ^a^ ± 0. 32	2.87 ^a^ ± 0.84
0.3 m/s	0.15 ^b^ ± 0.11	0.15 ^b^ ± 0.03	2.03 ^a^ ± 0.59	4.10 ^b^ ± 0.10

Different letters (a, b) in the same column indicate significant differences (*p* < 0.05).

## Data Availability

Some or all data, models, or code that support the findings of this study are available from the corresponding author upon reasonable request.
